# Association between stopping renin-angiotensin system inhibitors immediately before hemodialysis initiation and subsequent cardiovascular events

**DOI:** 10.1038/s41440-024-01616-8

**Published:** 2024-03-04

**Authors:** Yoshihiro Nakamura, Daijo Inaguma, Takahiro Imaizumi, Shimon Kurasawa, Manabu Hishida, Masaki Okazaki, Yuki Fujishima, Nobuhiro Nishibori, Katsuhiko Suzuki, Yuki Takeda, Shoichi Maruyama

**Affiliations:** 1 Department of Nephrology, Nagoya University Graduate School of Medicine, 65 Tsurumai-cho, Showa-ku, Nagoya, Aichi 466-8550 Japan; 2 Department of Internal Medicine, Fujita Health University Bantane Hospital, 3-6-10, Otobashi, Nakagawa-ku, Nagoya, Aichi 454-8509 Japan; 3 Department of Advanced Medicine, Nagoya University Hospital, 65 Tsurumai-cho, Showa-ku, Nagoya, Aichi 466-8550 Japan; 4 Department of Nephrology, Kaikoukai Josai Hospital, Nagoya, Japan, 4-1, Kitahata-cho, Nakamura-ku, Nagoya, Aichi 453-0815 Japan; 5 Department of Clinical Research Education, Nagoya University Graduate School of Medicine, 65 Tsurumai-cho, Showa-ku, Nagoya, Aichi 466-8550 Japan

**Keywords:** advanced chronic kidney disease, cardiovascular event, hemodialysis, renin-angiotensin system inhibitors

## Abstract

It is controversial whether renin-angiotensin system inhibitors (RASIs) should be stopped in patients with advanced chronic kidney disease (CKD). Recently, it was reported that stopping RASIs in advanced CKD was associated with increased mortality and cardiovascular (CV) events; however, it remains unclear whether stopping RASIs before dialysis initiation affects clinical outcomes after dialysis, which this study aimed to evaluate. In this multicenter prospective cohort study in Japan, we included 717 patients (mean age, 67 years; 68% male) who had a nephrology care duration ≥90 days, initiated hemodialysis, and used RASIs 3 months before hemodialysis initiation. The multivariable adjusted Cox models were used to compare mortality and CV event risk between 650 (91%) patients who continued RASIs until hemodialysis initiation and 67 (9.3%) patients who stopped RASIs. During a median follow-up period of 3.5 years, 170 (24%) patients died and 228 (32%) experienced CV events. Compared with continuing RASIs, stopping RASIs was unassociated with mortality (adjusted hazard ratio [aHR]: 0.82; 95% confidence interval [CI]: 0.50–1.34) but was associated with higher CV events (aHR: 1.59; 95% CI: 1.06–2.38). Subgroup analyses showed that the risk of stopping RASIs for CV events was particularly high in patients aged <75 years, with a significant interaction between stopping RASIs and age. This study revealed that patients who stopped RASIs immediately before dialysis initiation were associated with subsequent higher CV events. Active screening for CV disease may be especially beneficial for these patients.

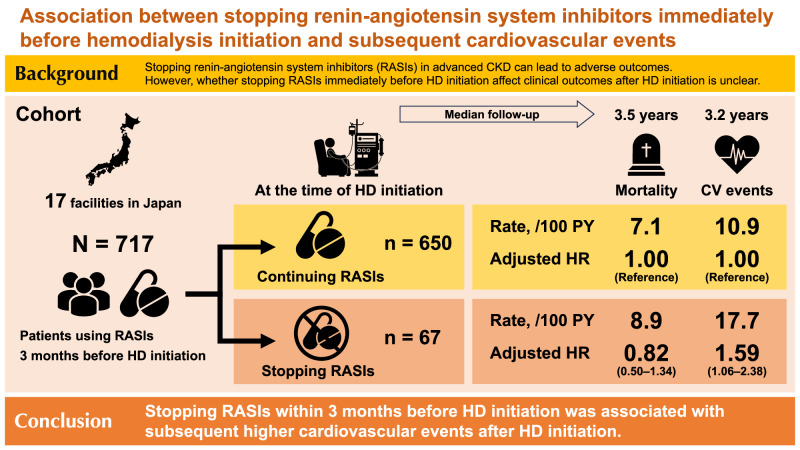

## Introduction

Chronic kidney disease (CKD) patients are at higher risk for cardiovascular (CV) events and mortality than the general population and require optimal management to reduce these risks [1, 2]. Renin-angiotensin system inhibitors (RASIs) have been reported to reduce the risk of kidney failure, CV events, and all-cause mortality in patients with CKD [3]. Thus, guidelines recommend using RASIs for advanced CKD (i.e., CKD stage 4 or 5) unless they are intolerable due to hyperkalemia or worsening of kidney function. However, the benefits of using RASIs for advanced CKD are less certain because most clinical trials excluded participants with advanced CKD [2, 4–6].

Several studies investigated whether RASIs should be stopped or continued in patients with advanced CKD [7–13]. Stopping RASIs in patients with advanced CKD was associated with all-cause mortality [8, 9] and CV events [8, 9, 11]. However, the observational period of these studies was until the initiation of dialysis; thus, whether stopping RASIs before dialysis initiation affects prognosis after the initiation of dialysis is uncertain.

This study aimed to evaluate whether stopping RASIs immediately before dialysis initiation affects subsequent outcomes, such as mortality and CV events, in patients registered with the Aichi cohort study of the prognosis in patients newly initiated into dialysis (AICOPP), which is a multicenter, prospective cohort study.

## Materials and methods

### Study population

We used data from the AICOPP, including 1520 incident dialysis patients. Details of the AICOPP have been previously described [14]. The cohort included patients who initiated dialysis between October 2011 and September 2013 at 17 facilities in Aichi, Japan. We screened patients aged ≥20 years and enrolled those who were discharged alive after hospitalization for dialysis initiation. Written informed consent was obtained from all patients. In our study of patients registered with AICOPP, we excluded patients referred to nephrologists <90 days prior to dialysis initiation or whose duration of nephrologist care was unknown, and patients who opted for peritoneal dialysis.

We recruited patients who had used angiotensin converting enzyme inhibitors (ACEIs) or angiotensin II receptor blockers (ARBs) 3 months prior to hemodialysis (HD) initiation and those who had data of ACEIs or ARBs at the time of HD initiation for survival analysis.

### Baseline variables

Baseline demographic and clinical data, including blood and urine test results, were collected immediately before or during hospitalization for HD initiation. Body mass index (BMI) was calculated using the following formula: BMI = weight(kg)/height(m)^2^. Diabetes mellitus was defined as fasting blood glucose ≥126 mg/dL, casual blood glucose ≥200 mg/dL, HbA1c (NGSP) ≥ 6.5%, use of insulin, or use of oral hypoglycemic agents. A history of CV disease (CVD) was defined as a history of heart failure requiring hospitalization, coronary artery intervention, heart bypass surgery, stroke, aortic disease requiring surgery, or peripheral artery disease requiring hospitalization. Urgent dialysis was defined as emergency dialysis or dialysis initiation using an indwelling vascular catheter when faced with a risk to life. Emergency dialysis initiation was referred to as unscheduled initiation. The estimated glomerular filtration rate (eGFR) was calculated using the Japanese Society of Nephrology’s equation: eGFR = 194 × serum creatinine^–1.094^ × age^–0.287^ (×0.739 for women). The use of diuretics before dialysis initiation included regular loop diuretics, thiazide-type diuretics, or spironolactone [14, 15]. The use of RASIs was defined as the use of ACEIs or ARBs. We divided patients into four groups according to the patterns of RASIs use 3 months before and at the time of HD initiation: continued, stopped, did not use, and started during the 3 months prior to HD initiation [16]. Patients with missing this information were assigned to the unknown group (Supplementary Table 1).

### Outcomes

The study outcomes were all-cause mortality and CV events after HD initiation. CV events were defined as heart failure, acute coronary syndrome, stroke, or peripheral artery disease requiring hospitalization [15]. Outcome data of all patients in this cohort were collected by reviewing the medical records of the AICOPP group or by sending letters to each dialysis clinic where patients were transferred for maintenance HD. Patients were followed up from the day of dialysis initiation, until either death, failure to follow-up, kidney transplantation, recovery from dialysis therapy, or the end of follow-up on September 30, 2016 [17].

### Statistical analysis

The baseline characteristics of the cohort for survival analysis were summarized into two groups: continuing RASIs and stopping RASIs, with normally and non-normally distributed variables and categorical variables as mean (standard deviation [SD]), median (interquartile range [IQR]), and number (percentage), respectively. Group differences were assessed using Student’s t test, Wilcoxon rank sum test, and chi-square test for continuous variables with approximately normal distributions, non-normally distributed variables, and categorical data, respectively.

We estimated survival across the two groups using the Kaplan–Meier method. Differences in survival estimates between the two groups were assessed using the log-rank test. We performed analyses using multiple imputations by chained equations in the multivariable models to handle missing data. We performed chained equations with 100 imputations and combined the estimates of the analysis per dataset using Rubin’s rule. After multiple imputations, we constructed the multivariable Cox proportional hazard models to assess the risk of mortality and CV events associated with stopping RASIs and clinical characteristics. Three models were explored: Model 1 was unadjusted; Model 2 was adjusted for age, sex, BMI, history of diabetes mellitus, and history of CVD; and Model 3 additionally accounted for serum creatinine, serum potassium, eGFR decline (the change in eGFR for 3 months before dialysis initiation), and use of ion exchange resin, antiplatelet drugs, β-blockers, diuretics, and urgent dialysis. As sensitivity analyses for CV events in the stopping RASIs group, we performed Fine and Gray competing-risk analyses considering non-CV death as a competing risk. In case of significant association between mortality or CV events, and stopping RASIs, subgroup analyses according to age (<75 vs. ≥75 years), sex, diabetes mellitus, and history of CVD were performed.

In all analyses, a two-sided *p*-value of <0.05 was considered statistically significant. All the statistical analyses were performed using Stata version 17 (StataCorp, College Station, Texas, USA).

## Results

### Patient characteristics

**Fig. 1:**
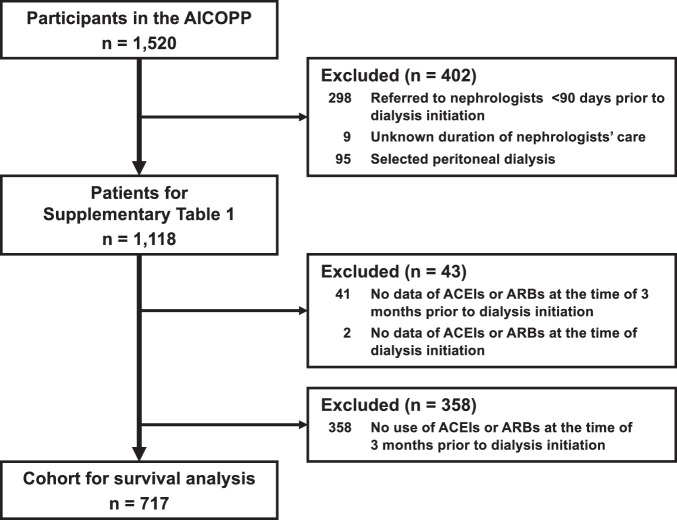
Flow diagram illustrating patient enrollment for the present study. A total of 1118 patients were included in the baseline data (Supplementary Table 1), and 717 in the survival analysis. ACEIs angiotensin-converting enzyme inhibitors, ARBs angiotensin II receptor blockers

Of the 1118 patients, 717 were included for the survival analysis (Fig. 1). Table 1 shows the baseline characteristics of the analytical cohorts in the two groups. The characteristics of the eligible patients were similar to those of patients excluded due to non-using, newly starting RASIs or unknown data (Supplementary Table 1). The mean ± SD age of 717 study participants at HD initiation was 67 ± 13 years, and 68% were male. The numbers of patients with continuing and stopping RASIs were 650 (91%) and 67 (9.3%), respectively. Patients who stopped RASIs had a shorter duration of nephrology care, more frequent urgent dialysis, faster eGFR decline, and higher serum C-reactive protein levels than those who continued RASIs. Missing data were found in ≤5% of all variables (Supplementary Table 2).

**Table 1:** Baseline characteristics by the study group

	Total	Continuing RASIs	Stopping RASIs
	N = 717	n = 650	n = 67
Clinical parameters			
Age, years	67 ± 13	67 ± 13	68 ± 14
Male sex	485 (68%)	441 (68%)	44 (66%)
Body mass index, kg/m^2^	24.2 ± 4.3	24.1 ± 4.2	24.7 ± 5.0
Underlying disease^a^			
Diabetic kidney disease	366 (51%)	333 (51%)	33 (49%)
Glomerulonephritis	116 (16%)	111 (17%)	5 (7.5%)
Nephrosclerosis	159 (22%)	138 (21%)	21 (31%)
Polycystic kidney disease	27 (3.8%)	27 (4.2%)	0 (0%)
Others	28 (3.9%)	26 (4.0%)	2 (3.0%)
Unknown	21 (2.9%)	15 (2.3%)	6 (9.0%)
Diabetes mellitus	407 (57%)	371 (57%)	36 (54%)
History of cardiovascular disease	314 (44%)	289 (45%)	25 (37%)
Charlson comorbidity index	5 (3–6)	5 (3–6)	5 (4–6)
Duration of nephrology care^a^, years	2.2 (1.1–4.2)	2.3 (1.1–4.3)	1.5 (0.8–3.3)
Smoking history	211 (53%)	193 (54%)	18 (44%)
Urgent dialysis^a^	164 (23%)	142 (22%)	22 (33%)
Vascular access			
Arteriovenous fistula	581 (81%)	533 (82%)	48 (72%)
Arteriovenous graft	54 (7.6%)	45 (7.0%)	9 (13%)
Temporary catheter	79 (11%)	69 (11%)	10 (15%)
Systolic blood pressure, mmHg	153 ± 25	154 ± 25	149 ± 28
Diastolic blood pressure, mmHg	77 ± 14	77 ± 14	78 ± 15
Laboratory measurements			
eGFR 3 months before dialysis initiation^a^, mL/min/1.73 m^2^	8.1 ± 3.3	7.9 ± 3.1	9.7 ± 4.0
eGFR at dialysis initiation^a^, mL/min/1.73 m^2^	5.3 ± 1.8	5.2 ± 1.8	5.7 ± 1.9
eGFR decline^a,b^, mL/min/1.73 m^2^	2.8 ± 2.7	2.6 ± 2.6	4.0 ± 3.3
Hemoglobin, g/dL	9.4 ± 1.4	9.4 ± 1.4	9.6 ± 1.5
Albumin^a^, g/dL	3.2 ± 0.6	3.2 ± 0.6	3.1 ± 0.6
Urea nitrogen, mg/dL	90.8 ± 27.7	91.2 ± 27.5	87.0 ± 29.5
Creatinine^a^, mg/dL	9.1 ± 2.9	9.1 ± 3.0	8.3 ± 2.5
Uric acid, mg/dL	8.6 ± 2.2	8.5 ± 2.2	9.0 ± 2.9
Potassium, mEq/L	4.6 ± 0.8	4.6 ± 0.8	4.4 ± 0.9
Corrected calcium, mg/dL	8.6 ± 1.1	8.6 ± 1.1	8.8 ± 1.0
Phosphorus, mg/dL	6.3 ± 1.7	6.3 ± 1.7	6.2 ± 2.0
Intact parathyroid hormone, pg/mL	313 (197–445)	316 (200–446)	263 (192–434)
C–reactive protein^a^, mg/dL	0.20 (0.09–1.14)	0.20 (0.09–1.01)	0.55 (0.17–2.29)
Low-density lipoprotein cholesterol, mg/dL	88 ± 32	87 ± 32	94 ± 33
Bicarbonate, mmol/L	19.7 ± 4.5	19.8 ± 4.4	19.4 ± 4.9
Medications			
ACEIs 3 months before dialysis initiation	96 (13%)	82 (13%)	14 (21%)
ARBs 3 months before dialysis initiation^a^	681 (95%)	624 (96%)	57 (85%)
ACEIs^a^	84 (12%)	84 (13%)	0 (0%)
ARBs^a^	625 (87%)	625 (96%)	0 (0%)
β-blockers	264 (37%)	241 (37%)	23 (34%)
Calcium channel blockers^a^	625 (87%)	574 (88%)	51 (76%)
Direct renin inhibitors	26 (3.6%)	26 (4.0%)	0 (0%)
Diuretics	543 (76%)	492 (76%)	51 (76%)
Spironolactone	36 (5.0%)	30 (4.6%)	6 (9.0%)
Ion exchange resins	256 (38%)	232 (37%)	24 (39%)
Sodium bicarbonate	368 (51%)	334 (51%)	34 (51%)
Active vitamin D drugs	201 (28%)	185 (28%)	16 (24%)
Phosphate binders	273 (38%)	248 (38%)	25 (37%)
Antiplatelet drugs	238 (33%)	220 (34%)	18 (27%)
Statins	315 (44%)	290 (45%)	25 (37%)

Values are expressed as mean ± SD or median (interquartile range) for continuous measures, and number (percent) for categorical measures

*RASIs* renin-angiotensin system inhibitors, *eGFR* estimated glomerular filtration rate, *ACEIs* angiotensin converting enzyme inhibitors, *ARBs* angiotensin II receptor blockers

^a^*p* < 0.05 for Student’s *t* test, Wilcoxon rank sum test, or chi-square test

^b^eGFR decline indicates change in eGFR for 3 months before dialysis initiation

### Associations of stopping RASIs with mortality and CV events

**Fig. 2:**
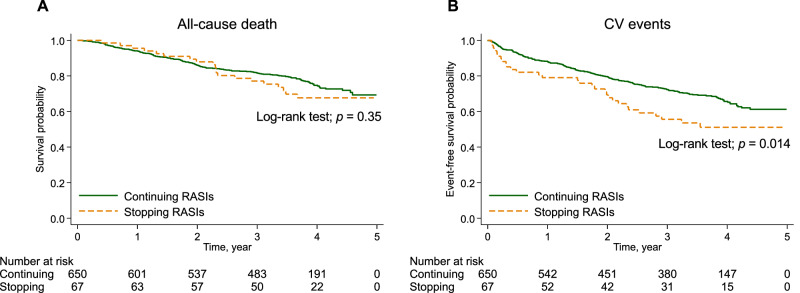
Time-to-event curves for study outcomes. The outcomes were all-cause death (**A**) and CV events (**B**). Stopping RASIs was significantly associated with a higher risk of CV events. RASIs renin-angiotensin system inhibitors, CV cardiovascular

The median [IQR] observation period of mortality was 3.5 [2.9–4.1] years, and 170 (24%) patients died. During follow-up, there were 74 (10%) censored cases comprising 15 patients who received kidney transplants and 59 patients who were lost to follow-up. Crude mortality rates (per 100 person-years) were 7.1 and 8.9 in patients who continued and stopped RASIs, respectively (Table 2). The Kaplan–Meier curves showed no difference in survival rates between the two groups. (*p* = 0.35; Fig. 2A). The multivariable analysis also showed no significant association between continuing or stopping RASIs and mortality; the multivariable-adjusted hazard ratios (HRs) was 0.82 (95% confidence interval [CI], 0.50–1.34).

**Table 2:** Incidence rates and hazard ratios for mortality and cardiovascular event

	Cases, n (%)	Rate, /100 PY	Hazard ratio (95% confidence interval)		
			Model 1	Model 2	Model 3
Mortality					
Continuing RASIs (n = 650)	150 (23%)	7.1	1.00 (Reference)	1.00 (Reference)	1.00 (Reference)
Stopping RASIs (n = 67)	20 (30%)	8.9	1.25 (0.78–1.99)	1.23 (0.77–1.98)	0.82 (0.50–1.34)
Cardiovascular events					
Continuing RASIs (n = 650)	198 (30%)	10.9	1.00 (Reference)	1.00 (Reference)	1.00 (Reference)
Stopping RASIs (n = 67)	30 (45%)	17.7	1.61 (1.10–2.37)	1.79 (1.22–2.64)	1.59 (1.06–2.38)

Model 1: unadjusted; Model 2: adjusted for age, sex, body mass index, diabetes mellitus, and history of cardiovascular disease; Model 3: adjusted for Model 2 + creatinine, potassium, eGFR decline (the change in eGFR for 3 months before dialysis initiation), ion exchange resin, antiplatelet drugs, β-blockers, diuretics, and urgent dialysis

*PY* person-years, *RASIs* renin-angiotensin system inhibitors, *eGFR* estimated glomerular filtration rate

The median [IQR] observation period of CV events was 3.2 [1.5–3.9] years, and 228 (32%) patients experienced CV events. Crude CV event rates (per 100 person-years) were 10.9 and 17.7 in patients who continued and stopped RASIs, respectively (Table 2). The Kaplan–Meier curves showed a decreased CV event-free rate in the stopping group (*p* = 0.014; Fig. 2B). In the multivariable analysis, stopping RASIs was associated with increased CV event risk; the multivariable-adjusted HRs was 1.59 (95% CI, 1.06–2.38). The results of the competing risk analysis were similar to those of the Cox regression analysis (subdistribution HR, 1.66; 95% CI, 1.05–2.62).

### Subgroup analyses

**Fig. 3:**
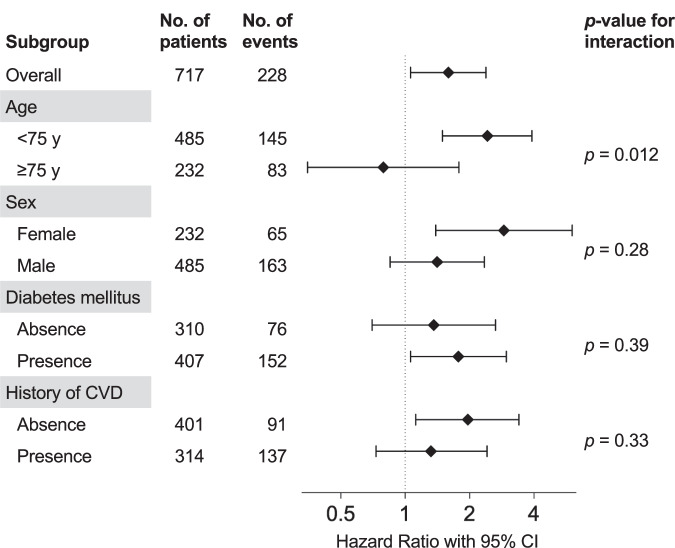
Subgroup analyses of CV events. The plots with capped spikes indicate the multivariable-adjusted hazard ratios with 95% confidence intervals for CV events of stopping RASIs compared with continuing RASIs. There was a significant interaction with relatively higher hazard ratios of CV events between stopping RASIs and age (<75 or ≥75 years). CVD cardiovascular disease, CV cardiovascular, RASIs renin-angiotensin system inhibitors

Although the association between stopping RASIs and CV events was mostly consistent across subgroups, only the age category (<75 vs. ≥75 years) had a significant interaction regarding CV events (*p* = 0.012); the risk of stopping RASIs was especially higher in patients with <75 years (Fig. 3).

## Discussion

In this multicenter, prospective cohort study of patients initiating HD, we assessed the association of stopping RASIs during the 3 months prior to HD initiation with mortality and CV events. We found that stopping RASIs was associated with higher risk of CV events after HD initiation and was not associated with mortality. The subgroup analysis for CV events revealed that the association was consistent except for the subgroup of age ≥75 years; CV risk of stopping RASIs was especially higher in the subgroup with age <75 years, with a significant interaction.

Several studies showed that stopping RASIs in advanced CKD was associated with mortality [8, 9] and CV events [8, 9, 11]. However, dialysis initiation was a censoring event in these studies [8, 9, 11]; thus, whether stopping RASIs immediately prior to dialysis initiation adversely affects prognosis after dialysis initiation remains uncertain. A previous multicenter randomized control study showed that stopping RASIs in patients with advanced CKD was not associated with long-term rate of eGFR decrease [10]. CV events and death were secondary outcomes, and they were similar in both the stopping and continuing RASIs groups. To our knowledge, this is the first study to evaluate the association between stopping RASIs before dialysis initiation on mortality and CV events after HD initiation. Patients who stopped RASIs within 3 months prior to HD initiation had higher CV event rates than those who continued RASIs. This result supports the guideline from China for pre-dialysis to continue RASIs in advanced CKD even just before dialysis initiation [18]. In the Evidence-based Clinical Practice Guideline for CKD from Japan, CVD screening at least before the initiation of dialysis is recommended; however, there is not enough evidence to suggest that universal CVD screening is beneficial [2]. Our results suggest that active screening for CVD may be especially beneficial for patients stopping RASIs immediately before dialysis initiation.

Regarding mortality, previous studies showed that stopping RASIs in advanced CKD was associated with mortality [8, 9], while some studies showed that it was not associated with mortality [10, 11]. In our study, there was no significant association between continuing or stopping RASIs and mortality. One possible explanation is that, since the stopping RASIs group is likely to experience a faster decline in eGFR and consequently an earlier initiation of dialysis, the analysis using the time of dialysis initiation as the baseline may introduce a “lead time bias” that may lead to a longer life expectancy.

There are limited studies on the influence of the stopping RASIs according to age in patients with advanced CKD. In an observational study of advanced CKD patients, compared with continuing RASIs, stopping RASIs had a higher CV events risk [9]. In their study, subgroup analysis was divided by age, and the CV events risk was compared between subgroup with age <70 years and ≥70 years. The subgroup with age <70 years who stopped RASIs tended to experience higher CV events rates; however, no significant interaction was observed between stopping RASIs and age. In our study, RASIs in the subgroup with age ≥75 years was not associated with CV events risk, whereas CV events risk of stopping RASIs was especially higher in the subgroup with age <75 years, with a significant interaction (*p* = 0.012; Fig. 3). Thus, the benefits of continuing RASIs may be limited for the older. However, further investigation is needed to draw conclusions.

This study had several limitations. First, because this was an observational study, we could not directly verify whether stopping RASIs within 3 months before HD initiation had a positive influence on increasing CV events after HD initiation. In our cohort, patients who stopped RASIs had a higher prevalence of urgent dialysis and rapid eGFR decline for 3 months before dialysis initiation (Table 1). However, several baseline characteristics, such as age, male sex, and history of CV events, were comparable between the two groups. Additionally, multivariable analysis was performed to eliminate the influence of potential confounders as much as possible. Nevertheless, the influence of unmeasured confounders may remain. Second, very little information was available 3 months before HD initiation. In this regard, we adjusted for eGFR decline 3 months before dialysis initiation, potassium level at HD initiation, potassium binder prescription at HD initiation, and urgent dialysis in the multivariate analysis; however, information regarding the reason for stopping RASIs was unavailable. For example, although hyperkalemia within 3 months of starting dialysis may have led to stopping RASIs, data on potassium levels at 3 months before HD initiation were not collected. Furthermore, CV events immediately before dialysis initiation could prompt stopping RASIs; however, information on the timing of the history of CV events was also not collected. Third, we could identify information on RASIs use only at 3 months before dialysis initiation and at the time of dialysis initiation. Fourth, the sample size was relatively small (stopping RASIs group, *n* = 67).

In conclusion, patients who stopped RASIs within 3 months before HD initiation had a higher risk of CV events after HD initiation. Active screening for CVD may be especially beneficial for these patients.

### Supplementary information

Supplementary Material
