# Supplementary material for: Association between stopping renin-angiotensin system inhibitors immediately before hemodialysis initiation and subsequent cardiovascular events
Source: Hypertens Res. 2024 Mar 4;47(5):1372–9. doi: 10.1038/s41440-024-01616-8 (PMC11073956; doi:10.1038/s41440-024-01616-8)
Supplement: Supplementary file 1 — Supplementary Material [file 41440_2024_1616_MOESM1_ESM.pdf]

Supplementary Material for:

Association between stopping renin-angiotensin system inhibitors immediately before hemodialysis initiation and subsequent cardiovascular events

Table of Contents:

Supplementary Table 1. Baseline characteristics based on the patterns of RASIs use 3 months before and at the time of hemodialysis initiation 2

Supplementary Table 2. Number and percentage of missing data in patients for survival analysis 3

Supplementary Table 1. Baseline characteristics based on the patterns of RASIs use 3 months before and at the time of hemodialysis initiation

	Total N = 1,118	Continuing n = 650	Stopping n = 67	None-using n = 311	Starting n = 47	Unknown n = 43
**Clinical parameters**						
Age, years	68 ± 13	67 ± 13	68 ± 14	71 ± 12	65 ± 13	69 ± 9
Male sex	764 (68%)	441 (68%)	44 (66%)	211 (68%)	36 (77%)	32 (74%)
Body mass index, kg/m^2	23.7 ± 4.3	24.1 ± 4.2	24.7 ± 5.0	22.6 ± 4.3	23.4 ± 4.6	23.6 ± 3.4
Underlying disease						
Diabetic kidney disease	524 (47%)	333 (51%)	33 (49%)	122 (39%)	19 (40%)	17 (40%)
Glomerulonephritis	159 (14%)	111 (17%)	5 (7.5%)	32 (10%)	4 (8.5%)	7 (16%)
Nephrosclerosis	287 (26%)	138 (21%)	21 (31%)	101 (32%)	15 (32%)	12 (28%)
Polycystic kidney disease	40 (3.6%)	27 (4.2%)	0 (0%)	9 (2.9%)	2 (4.3%)	2 (4.7%)
Others	66 (5.9%)	26 (4.0%)	2 (3.0%)	31 (10%)	4 (8.5%)	3 (7.0%)
Unknown	42 (3.8%)	15 (2.3%)	6 (9.0%)	16 (5.1%)	3 (6.4%)	2 (4.7%)
Diabetes mellitus	606 (54%)	371 (57%)	36 (54%)	148 (48%)	27 (57%)	24 (56%)
History of cardiovascular disease	515 (46%)	289 (45%)	25 (37%)	156 (50%)	24 (51%)	21 (49%)
Charlson comorbidity index	5 (3–6)	5 (3–6)	5 (4–6)	5 (4–6)	5 (4–6)	4 (3–5)
Duration of nephrology care, years	2.1 (1.0–4.3)	2.3 (1.1–4.3)	1.5 (0.8–3.3)	1.9 (0.9–4.4)	1.6 (0.8–4.0)	2.6 (1.1–5.7)
Smoking history	364 (57%)	193 (54%)	18 (44%)	119 (62%)	19 (66%)	15 (62%)
Urgent dialysis	273 (25%)	142 (22%)	22 (33%)	86 (28%)	14 (30%)	9 (21%)
Vascular access						
Arteriovenous fistula	883 (79%)	533 (82%)	48 (72%)	229 (74%)	39 (83%)	34 (79%)
Arteriovenous graft	80 (7.2%)	45 (7.0%)	9 (13%)	21 (6.8%)	1 (2.1%)	4 (9.3%)
Temporary catheter	149 (13%)	69 (11%)	10 (15%)	58 (19%)	7 (15%)	5 (12%)
Systolic blood pressure, mmHg	153 ± 26	154 ± 25	149 ± 28	151 ± 26	156 ± 22	151 ± 27
Diastolic blood pressure, mmHg	77 ± 14	77 ± 14	78 ± 15	77 ± 15	82 ± 14	77 ± 16
**Laboratory measurements**						
eGFR 3 months before dialysis initiation, mL/min/1.73 m^2	8.2 ± 3.4	7.9 ± 3.1	9.7 ± 4.0	8.5 ± 3.9	8.4 ± 2.7	7.8 ± 2.3
eGFR at dialysis initiation, mL/min/1.73 m^2	5.3 ± 1.9	5.2 ± 1.8	5.7 ± 1.9	5.5 ± 2.0	5.5 ± 1.6	4.9 ± 1.4
eGFR decline[a], mL/min/1.73 m^2	2.8 ± 2.8	2.6 ± 2.6	4.0 ± 3.3	3.0 ± 3.1	2.9 ± 2.7	2.6 ± 2.0
Hemoglobin, g/dL	9.4 ± 1.4	9.4 ± 1.4	9.6 ± 1.5	9.4 ± 1.4	10.0 ± 1.4	9.0 ± 1.6
Albumin, g/dL	3.2 ± 0.6	3.2 ± 0.6	3.1 ± 0.6	3.2 ± 0.6	3.4 ± 0.5	3.4 ± 0.6
Urea nitrogen, mg/dL	91.7 ± 28.1	91.2 ± 27.5	87.0 ± 29.5	93.7 ± 30.0	89.3 ± 25.5	95.8 ± 23.5
Creatinine, mg/dL	8.9 ± 2.9	9.1 ± 3.0	8.3 ± 2.5	8.6 ± 2.7	9.0 ± 3.4	9.4 ± 2.3
Uric acid, mg/dL	8.6 ± 2.3	8.5 ± 2.2	9.0 ± 2.9	8.7 ± 2.5	9.4 ± 2.0	8.2 ± 1.9
Potassium, mEq/L	4.5 ± 0.8	4.6 ± 0.8	4.4 ± 0.9	4.4 ± 0.8	4.3 ± 0.5	4.7 ± 0.7
Corrected calcium, mg/dL	8.6 ± 1.0	8.6 ± 1.1	8.8 ± 1.0	8.6 ± 1.0	8.9 ± 0.8	8.7 ± 0.9
Phosphorus, mg/dL	6.3 ± 1.7	6.3 ± 1.7	6.2 ± 2.0	6.2 ± 1.7	6.1 ± 1.9	6.0 ± 1.6
Intact parathyroid hormone, pg/mL	300 (191–437)	316 (200–446)	263 (192–434)	285 (184–410)	250 (149–399)	293 (188–559)
C-reactive protein, mg/dL	0.24 (0.10–1.24)	0.20 (0.09–1.01)	0.55 (0.17–2.29)	0.35 (0.10–1.48)	0.20 (0.03–0.63)	0.34 (0.20–0.94)
Low-density lipoprotein cholesterol, mg/dL	88 ± 33	87 ± 32	94 ± 33	90 ± 35	95 ± 39	84 ± 34
Bicarbonate, mmol/L	19.9 ± 4.6	19.8 ± 4.4	19.4 ± 4.9	19.9 ± 5.0	21.4 ± 4.6	20.0 ± 4.7
**Medications**						
ACEIs 3 months before dialysis initiation	97 (9.0%)	82 (13%)	14 (21%)	0 (0%)	0 (0%)	1 (50%)
ARBs 3 months before dialysis initiation	682 (63%)	624 (96%)	57 (85%)	0 (0%)	0 (0%)	1 (33%)
ACEIs	104 (9.3%)	84 (13%)	0 (0%)	0 (0%)	13 (28%)	7 (16%)
ARBs	690 (62%)	625 (96%)	0 (0%)	0 (0%)	35 (74%)	30 (73%)
β-blockers	412 (37%)	241 (37%)	23 (34%)	109 (35%)	20 (43%)	19 (44%)
Calcium channel blockers	918 (82%)	574 (88%)	51 (76%)	227 (73%)	31 (66%)	35 (81%)
Direct renin inhibitors	46 (4.1%)	26 (4.0%)	0 (0%)	13 (4.2%)	2 (4.3%)	5 (12%)
Diuretics	837 (75%)	492 (76%)	51 (76%)	232 (75%)	25 (53%)	37 (86%)
Spironolactone	60 (5.4%)	30 (4.6%)	6 (9.0%)	21 (6.8%)	3 (6.4%)	0 (0%)
Ion exchange resins	361 (36%)	232 (37%)	24 (39%)	96 (34%)	8 (18%)	1 (25%)
Sodium bicarbonate	547 (49%)	334 (51%)	34 (51%)	141 (45%)	16 (34%)	22 (51%)
Active vitamin D drugs	318 (28%)	185 (28%)	16 (24%)	90 (29%)	12 (26%)	15 (35%)
Phosphate binders	420 (38%)	248 (38%)	25 (37%)	119 (38%)	11 (23%)	17 (40%)
Antiplatelet drugs	370 (33%)	220 (34%)	18 (27%)	100 (32%)	17 (36%)	15 (35%)
Statins	485 (43%)	290 (45%)	25 (37%)	129 (41%)	17 (36%)	24 (56%)

Values are expressed as mean ± SD or median (interquartile range) for continuous measures, and number (percent) for categorical measures.

[a] eGFR decline indicates the change in eGFR for 3 months before dialysis initiation.

RASIs, renin-angiotensin system inhibitors; eGFR, estimated glomerular filtration rate; ACEIs, angiotensin converting enzyme inhibitors; ARBs, angiotensin II receptor blockers.

Supplementary Table 2. Number and percentage of missing data in patients for survival analysis (n = 717)

Variables	n (%)
Age	0 (0%)
Sex	0 (0%)
Body mass index	9 (1.3%)
Diabetes mellitus	0 (0%)
History of cardiovascular disease	2 (0.3%)
Creatinine	0 (0%)
Potassium	0 (0%)
eGFR decline[a]	22 (3.1%)
Ion exchange resins use	36 (5.0%)
Antiplatelet drugs use	0 (0%)
β-blockers use	0 (0%)
Diuretics use	0 (0%)
Urgent dialysis	3 (0.4%)

[a] eGFR decline indicates the change in eGFR for 3 months before dialysis initiation.

eGFR, estimated glomerular filtration rate.
